# The Neo-Malthusian League

**Published:** 1914-11

**Authors:** 


### The Neo-Malthusian League.

This league is now pretty well established in most of the countries around the globe. It has for its object the laudable purpose of abolishing poverty and suffering in society, and the betterment of the human species. In this respect, the league is a valuable handmaiden in the larger field of eugenics. It proposes to accomplish its object by the deliberate limitation of the family, and furnishes to its members the information necessary to accomplish this purpose in a hygienic and harmless manner. It encourages early marriages and the family unit as being most conducive in the highest degree to family happiness and hygienic living. But, for the sake of the child, the mother and the father, the number of children born should be no more than the parents are able to provide for, mentally, physically and morally, which includes prenatal consideration for the mother. In this respect, the league differs from the proposition of Malthus, who favored late marriages or celibacy, hence the name "neo-malthusian."

The league assumes the principle that the death rate falls with the constant fall in birth rate, and that population has a constant tendency to increase beyond the means of subsistence, unless consciously and sufficiently controlled. On this presumption, the league regards it as being more moral and humane to prevent conception than to destroy it, and eminently more consistent with the plan of nature to encourage early marriages, that the sex instinct may be normally conserved.

This attitude appeals to most thinking men, and their only serious objection to its open practice, no doubt, is the deep-seated mores against the prevention of conception. But we must remember that advancement, whether it be material or physical, is evolutional, and this change in custom is but the result of this unalterable, universal law. Already, the better-to-do classes are restricting their families of their own volition, while the greatest birth rate is to be found among those who are the least able and fitted for the responsibility. Herein lies the justification for a concert of action in regulating this matter. But to those of us who live in the United States, we are debarred by our national laws from engaging in any open crusade of this sort. To practice, or to offer any means to others for the prevention of conception, is regarded as a crime against society, and the State so heinous as to demand the severest punishment (five years in the penitentiary and five thousand dollars' fine). This statute stands as the will of the people, and will remain as such until the will of the people decree otherwise.

Whether the mission of this league deserves your support is a matter of personal conviction. It will be, undoubtedly, regardless of contrary beliefs, a very potent factor in determining the character and destiny of the next generation. If you believe these principles to be right, and to be to the best interest of society, then you should openly declare yourself, to the end that public opinion will demand the repeal of the laws that prohibit their fulfillment.

